# Co-circulation and persistence of multiple A/H3N2 influenza variants in China

**DOI:** 10.1080/22221751.2019.1648183

**Published:** 2019-08-02

**Authors:** Weifeng Shi, Changwen Ke, Shisong Fang, Juan Li, Hao Song, Xiyan Li, Tao Hu, Jie Wu, Tao Chen, Lina Yi, Yingchao Song, Xin Wang, Weijia Xing, Weijuan Huang, Hong Xiao, Lijun Liang, Bo Peng, Weihua Wu, Hui Liu, William J. Liu, Edward C. Holmes, George F. Gao, Dayan Wang

**Affiliations:** a Chinese National Influenza Center, National Institute for Viral Disease Control and Prevention, Chinese Center for Disease Control and Prevention, Beijing, People’s Republic of China; b WHO Collaborating Center for Reference and Research on Influenza, Beijing, People’s Republic of China; c Key Laboratory for Medical Virology, National Health Commission, Beijing, People’s Republic of China; d Key Laboratory of Etiology and Epidemiology of Emerging Infectious Diseases in Universities of Shandong, Shandong First Medical University & Shandong Academy of Medical Sciences, Taian, People’s Republic of China; e Guangdong Provincial Center for Disease Control and Prevention, Guangzhou, People’s Republic of China; f Division of Microbiology Test, Shenzhen Centre for Disease Control and Prevention, Shenzhen, People’s Republic of China; g Chinese Academy of Sciences, Research Network of Immunity and Health (RNIH), Beijing Institutes of Life Science, Beijing, People’s Republic of China; h Guangdong Provincial Institution of Public Health, Guangdong Provincial Center for Disease Control and Prevention, Guangzhou, People’s Republic of China; i Marie Bashir Institute for Infectious Diseases and Biosecurity, Charles Perkins Centre, School of Life and Environmental Sciences and Sydney Medical School, The University of Sydney, Sydney, Australia; j Key Laboratory of Pathogenic Microbiology and Immunology, Institute of Microbiology, Chinese Academy of Sciences, Beijing, People’s Republic of China; k Center for Influenza Research and Early-Warning (CASCIRE), Chinese Academy of Sciences, Beijing, People’s Republic of China; l Chinese Center for Disease Control and Prevention (China CDC), Beijing, People’s Republic of China

**Keywords:** Influenza virus, A/H3N2, vaccine, mutation

## Abstract

The spread of influenza A/H3N2 variants possessing the hemagglutinin 121 K mutation and the unexpectedly high incidence of influenza in the 2017–2018 northern hemisphere influenza season have raised serious concerns about the next pandemic. We summarized the national surveillance data of seasonal influenza in China and identified marked differences in influenza epidemics between northern and southern China, particularly the predominating subtype and the presence of an additional summer peak in southern China. Notably, a minor spring peak of influenza caused by a different virus subtype was also observed. We also revealed that the 3C.2a lineage was dominant from the summer of 2015 to the end of the 2015–2016 peak season in China, after which the 3C.2a2 lineage predominated despite the importation and co-circulation of the 121 K variants of 3C.2a1 and 3C.2a3 lineages at the global level. Finally, an analysis based on genetic distances revealed a delay in A/H3N2 vaccine strain update. Overall, our results highlight the complicated circulation pattern of seasonal influenza in China and the necessity for a timely vaccine strain update worldwide.

## Introduction

Influenza activity differs between southern and northern China, with two peaks (winter and summer) in southern China and only a single peak (winter) in northern China [1]. However, between May and August of 2017, the abrupt increase in hospitalization fatality rates caused by seasonal influenza A/H3N2 in Hong Kong Special Administrative region of China received broad media coverage worldwide, leading to serious public health concerns [2]. Most striking was that the reported number of influenza cases in the 2017–2018 influenza season ranked second in China after the 2009 pandemic H1N1 [3].

Seasonal human A/H3N2 influenza virus is characterized by periodic and rapid antigenic variation [4–6] that may reduce vaccine effectiveness. Since the 2016–2017 influenza season in the northern hemisphere, a number of novel subclades have been detected during routine influenza surveillance worldwide [7,8]. In particular, a novel genetic variant with a N121 K mutation in the hemagglutinin (HA) protein emerged in Hong Kong and comprised more than 35% of the A/H3N2 viruses tested in May 2017 [2]. This mutation was located in epitope D, and variants possessing this mutation were reported to have a less vaccine effectiveness in Denmark in the 2016–2017 influenza season [9]. More importantly, a recent study suggested that most of the global population would be susceptible to the 121 K variants [10]. To date, this variant has been widely spread across Europe [7,9,11], Asia [2,8,12] and North America [13].

To provide a more comprehensive study of the genetic diversity and antigenic variation of A/H3N2, and to trace the origin and spread of the novel 121 K variant in China, we present a description of the national surveillance data of influenza-like illnesscombined with the sequence data of 1471 full-length genomes of A/H3N2 influenza A virus isolated across China since 2015.

## Materials and methods

### Sample collection

Throat or nasal swabs were collected from outpatients of the sentinel hospitals, with clinical evidence of influenza-like illness (ILI), defined as a person with sudden onset of fever ≥ 38°C and cough or sore throat. Samples collected at sentinel sites were sent to the network laboratories within 48 h. Real-time PT-PCR was performed to determine the influenza positive samples as well as the type and subtype of the influenza virus. The viruses were isolated with Madine-Darby canine kidney (MDCK) cells or embryonated chicken eggs by the network laboratories in a biosafety level 2 facility. Influenza positive isolates were sent to provincial CDC and the Chinese National Influenza Centre for additional laboratory tests.

### RNA Extraction, RT-PCR and sequencing

Viral RNA was extracted from 200 μl of the infected allantoic fluid of embryonated chicken egg or cell culture supernatant with QIAamp viral RNA mini kit (Qiagen, Inc.). For each virus strain, the RNA was eluted in 50 µl nuclease-free water. Complementary DNA was synthesized by reverse transcription reaction, and gene amplification by PCR was performed, whole genome sequencing of influenza A virus [14] was performed on the MiSeq high-throughput sequencing platform (Illumina, Inc., San Diego, CA, USA).This resulted in the generation of full-length genome sequences of 1417 influenza A/H3N2 viruses isolated across China since 2015.

### Phylogenetic analysis

Full-length genome sequences of human A/H3N2 influenza viruses isolated since 2015 were downloaded from the Influenza Virus Resource at the National Center for Biotechnology Information (NCBI) (http://www.ncbi.nlm.nih.gov/genomes/FLU/FLU.html) and the GISAID database on 30 November 2017. Repetitive sequences in the two databases were removed by matching strain names using in-house scripts. Sequences of low quality were also removed. The remaining sequences were combined with those generated in the present study. Eight datasets corresponding to the eight gene segments of influenza A virus were first aligned using Muscle [15] and then adjusted manually in Bioedit [16]. Phylogenetic analysis of the aligned HA and neuraminidase (NA) datasets were performed using RAxML [17] under the GTRGAMMA nucleotide substitution model [18] and with random starting trees. To assess nodal support 1000 bootstrap replicates were performed and all other parameters were set to default. Trees were visualized using FigTree (http://tree.bio.ed.ac.uk/software/figtree/).

To investigate the potential origin of the Chinese 121 K variants, the HA genes of global 121 K variants were downloaded from the GISAID database. These dataset comprised 10,088 HA gene sequences, including 165 from China, and was aligned using Muscle and manually adjusted using Bioedit. We used multidimensional scaling (MDS)was to analyze the relationships among the sequences by computing a distance matrix among the sequence dataset. We employed MDS rather than phylogentic trees as a visualization tool because of the very large size of the data set. Indeed, MDS has been previously used to visualize genome evolution [19] as well as antigenic variation of influenza viruses [4]. To reduce the computational burden, we estimated the pairwise sequence for the complete dataset distance matrix using Phylip [20]: this was used the input in the MDS analysis to identify the major groupings among these sequences. Based on the MDS analysis, the complete dataset was then sub-divided into two smaller sub-datasets. We then repeated the phylogenetic analysis described above on these sub-datasets using the same parameters, but with 100 bootstrap replicates. The trees generated in this case were also visualized using FigTree.

To help assess vaccine effectiveness and the timing of vaccine strain update, pairwise HA1 protein distances between vaccine strains and the circulating strains were also calculated using Phylipand visualized using in-house scripts.

### Antigenic variation analysis of H3 HAs

The crystal structure of A/H3N2 HA (A/Victoria/361/2011; PDB code:4O5N) was used to show the position of the A-E epitopes in H3 HA surface as antigenic sites. Compared with the HA of A/Victoria/361/2011, distinct residues which led to antigenic variation from six representative A/H3N2 virus strains were highlighted. The six HA sequences included were: (i) A/Texas/50/2012 (3C.1), (ii) A/Switzerland/9715293/2013 (3C.3a), (iii) A/HongKong/4801/2014 (3C.2a), (iv) A/Singapore/INFIMH160019/2016 (3C.2a1), (v) A/Guangdong/Dongguan_F20161100/2016 (3C.2a2), and (vi) A/Yunnan/Jinghong_1653/2017 (3C.2a3). Structure figures were visualized using PyMOL (http://pymol.org/).

## Results

### Epidemiological surveillance and distribution of the samples

According to the “Technical Manual of Chinese National Influenza Surveillance,” a virus is considered to be from northern China if it comes from 16 provinces (autonomous regions and municipalities): Beijing, Tianjin, Hebei, Shanxi, Inner Mongolia, Liaoning, Jilin, Heilong Jiang, Shandong, Henan, Tibet, Shaanxi, Gansu, Ningxia, Qinghai, and Xinjiang (Figure S1). Similarly, viruses from southern China are assigned from 15 provinces (autonomous regions and municipalities): Shanghai, Jiangsu, Zhejiang, Anhui, Fujian, Jiangxi, Hubei, Hunan, Guangdong, Guangxi, Hainan, Chongqing, Sichuan, Guizhou, and Yunnan (Figure S1).

**Figure 1.**
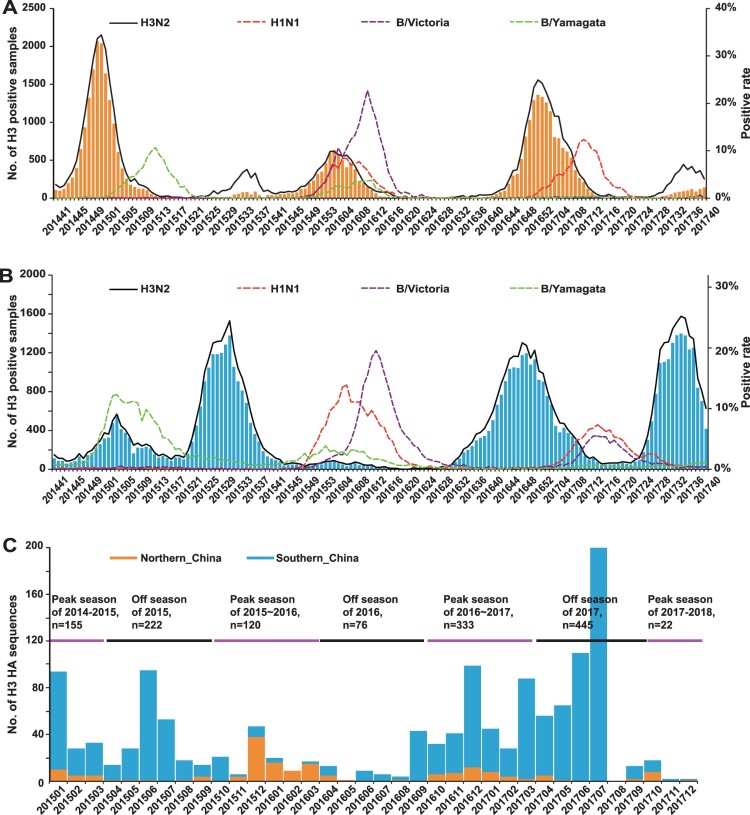
Epidemiological surveillance of seasonal human influenza in China since October 2014. Panels A and B show the epidemiological surveillance data of seasonal human influenza in northern and southern China since October 2014, respectively. In panels A and B, the bars represent the number of H3 positive samples, while the curves represent the positive rates of A/H3N2, A/H1N1, B/Yamagata and B/Victoria. Panel C shows the distribution of the 1417 strains newly sequenced in the present study.

Based on the ILI surveillance data obtained from Chinese National Influenza Centre, we found there was a clear seasonality of A/H3N2 activity both in northern (Figure 1A) and southern China (Figure 1B). However, a single influenza peak typically occurring during winter months was observed in northern China, and the positive rate of A/H3N2 was high in the 2014–2015 and 2016–2017 influenza seasons (Figure 1A). In the 2015–2016 influenza season, the B/Victoria lineage dominated over other subtypes in northern China. In particular, a second minor peak was observed in February following the earlier major A/H3N2 peak, as well as in the 2014–2015 and 2016–2017 influenza seasons which was caused by a different virus (the B/Yamagata lineage in 2015;A/H1N1 in 2017) (Figure 1A).

In contrast to the situation in northern China, influenza epidemics in southern China tended to have one summer peak and one winter peak (Figure 1B). The summer peak was clearly observed in 2015 and 2017 with A/H3N2 being the dominant subtype, but not observed in 2016, probably caused by the delayed end of the B/Victoria epidemics originating in early 2016. A/H3N2 also dominated over other subtypes in the 2016–2017 influenza season, which began in August, approximately eight weeks earlier than usual (Figure 1B). In particular, like northern China, a minor spring peak was observed in southern China in 2016 and 2017 (Figure 1B).

Southern China differs from northern China in a number of other aspects (Figure 1A and B). First, compared to other influenza seasons, the influenza activity was moderate in southern China in the 2014–2015 influenza season with B/Yamagata the dominant subtype (Figure 1B), unlike northern China in which A/H3N2 dominated (Figure 1A). Second, while A/H3N2 incidence was lowest in the 2015–2016 influenza season in southern China, this season ranked second in northern China.

### Genetic diversity of A/H3N2 in China during 2015–2017

To study the genetic diversity of A/H3N2 in China, a total of 1417 influenza A/H3N2 viruses isolated since 2015 were sequenced, including 172 from northern China and 1245 from southern China (948 from Guangdong province) (Figures 1C and S1). Our samples covered three winter seasons (2014–2015, 2015–2016 and 2016–2017) and three summer seasons (2015, 2016 and 2017) (Figure 1C). In addition, these strains were isolated from 15 of 16 provinces (autonomous regions and municipalities) in northern China (*n *= 172) and 14 of 15 provinces (autonomous regions and municipalities) in southern China (*n* = 1239), as well as in the Hong Kong and Macao Special Administration Region (*n *= 6) (Figure S1).

Based on the HA phylogeny (Supplementary file 1) and the specific amino acid substitutions, we determined the genotypes of the A/H3N2 viruses (*n *= 1655) circulating in China (Table 1). The majority of the strains (*n *= 1407, 85.02%) belonged to genotype 3C.2a (representative strain: A/HongKong/4801/2014) and its derived subclades. Among these, two subclades possessing the 121 K amino acid substitution were notable - 3C.2a1 (*n* = 103; representative strain: A/Singapore/INFIMH160019/2016) and 3C.2a3 (*n* = 45; representative strain: A/Yunnan/Jinghong_1653/2017). With one exception, 102 strains of 3C.2a1 possessed an additional N171 K mutation in HA1 and I77 V and G155E mutations in HA2, while all 3C.2a3 viruses possessed additional S144 K mutation in HA1 [7]. Apart from 3C.2a1 and 3C.2a3, there were other subclades derived from 3C.2a, one of which was 3C.2a2 (representative strain: A/Guangdong/Dongguan_F20161100/2016). This subclade was characterized of T131 K and R142 K in the HA protein. In addition, 188 strains (11.36%) belonged to genotype 3C.3a (representative strain: A/Switzerland/9715293/2013) and the remaining strains belonged to some minor genotypes, such as3C (*n* = 11), 3C.1 (*n* = 15; representative strain: A/Texas/50/2012), 3C.3 (*n* = 21), and 3C.3b (*n *= 9) (Table 1).

**Table 1.** Temporal distribution of the HA genotypes of influenza A/H3N2 virus in China.

Collection date		Clade 1	Clade 3C	Clade 3C.1	Clade 3C.2	Clade 3C.2a	Clade 3C.2a1	Clade 3C.2a2	Clade 3C.2a3	Clade 3C.3	Clade 3C.3a	Clade 3C.3b	Total
2014–2015 Peak season	Southern China		8	1	1	50				11	83	6	160
	Northern China					3				4	17		24
2015 Off-season	Southern China		1			250		1		1	19	2	274
	Northern China					11					1		12
2015–2016 Peak season	Southern China	2	2	2	1	53	1			1	15	1	78
	Northern China					146		1			27		174
2016 Off-season	Southern China			1		20		48			1		70
	Northern China					7		1					8
2016–2017 Peak season	Southern China			5		31	7	243	2	3	14		305
	Northern China					3		53			4		60
2017 Off-season	Southern China			6		40	90	283	32	1	6		458
	Northern China					4	1	4	1				10
2017–2018 Peak season	Southern China						1	4	7		1		13
	Northern China						3	3	3				9
Total	2	11	15	2	618	103	641	45	21	188	9	1655	

Remarkably, 3C.3a continued to circulate across multiple influenza seasons. Apart from the 2014–2015 peak season during which 3C.3a was the dominant genotype both in southern and northern China, 3C.2a was the dominant genotype in the influenza “off-season” of 2015 and in the 2015–2016 influenza season in China (Table 1). From the off-season of 2016 to that of 2017, 3C.2a2 predominated, although 3C.2a1 and 3C.2a3had emerged and begun to spread in China (Table 1).

**Figure 2.**
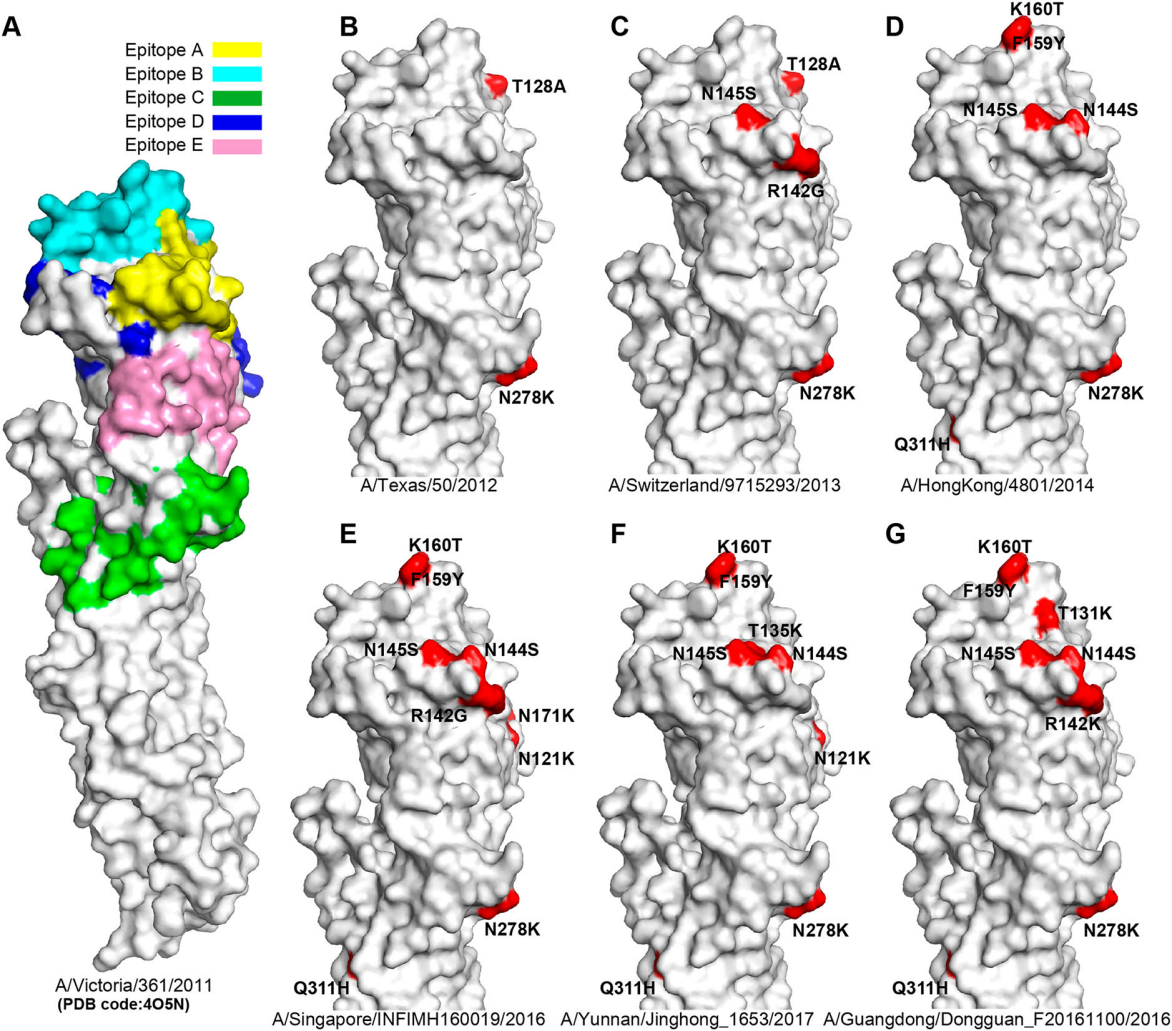
Antigenic variation analysis of H3 HAs. Panel A, the five antigenic sites (epitopes A-E) are shown in H3 HA surface. The monomer of A/H3N2 HA (A/Victoria/361/2011, PDB code:4O5N) is shown. Antigenic sites A (yellow), B (cyan), C (green), D (blue) and E (pink) are coloured as indicated. The HAs from representative strains A/Texas/50/2012 (B), A/Switzerland/9715293/2013 (C), A/HongKong/4801/2014 (D), A/Singapore/INFIMH160019/2016 (E), A/Yunnan/Jinghong_1653/2017 (F), A/Guangdong/Dongguan_F20161100/2016 (G) are compared with A/Victoria/361/2011, and the distinct amino acid substitutions which lead to antigenic variation are highlighted.

Consistent with the genetic analysis, homology modelling showed that the Chinese A/H3N2 viruses have evolved rapidly in the known epitopes during this time period (Figure 2). For example, compared to the vaccine strain A/HongKong/4801/2014(Figure 2D), A/Singapore/INFIMH160019/2016(representative strain of clade 3C.2a1) possessed the distinct N121 K and N171 K amino acid substitutions in epitope D, whereas A/Yunnan/Jinghong_1653/2017(representative strain of clade 3C.2a3) obtained a T135Ksubstitution in epitope A and N121 K in epitope D (Figure 2F), In addition, A/Guangdong/Dongguan_F20161100/2016 (representative strain of clade 3C.2a2, Figure 2G) possessed a T131 K and aR142 K in epitope A. Further study on the effects of specific amino acid substitutions at these antigenic sites may have important implications for improved vaccine development.

Similarly, based on our phylogenetic analysis (Supplementary file 2) and two recent reports [21,22], we also determined the NA genotypes of these viruses (Table S1). In the 2014–2015 influenza season, the dominant genotype was 3C.3, which continued to circulate during our surveillance. Subsequently, 3C.2a dominated in the 2015 off-season. It was then replaced by 3C.2b as the dominant genotype in the 2015–2016 influenza season in northern China. Although there was a dominant genotype in each influenza season, 3C.2a, 3C.2b and 3C.3 have co-circulated in China since 2015 (Table S1).

### The HA1 sequence divergence between vaccine and circulating strains

**Figure 3.**
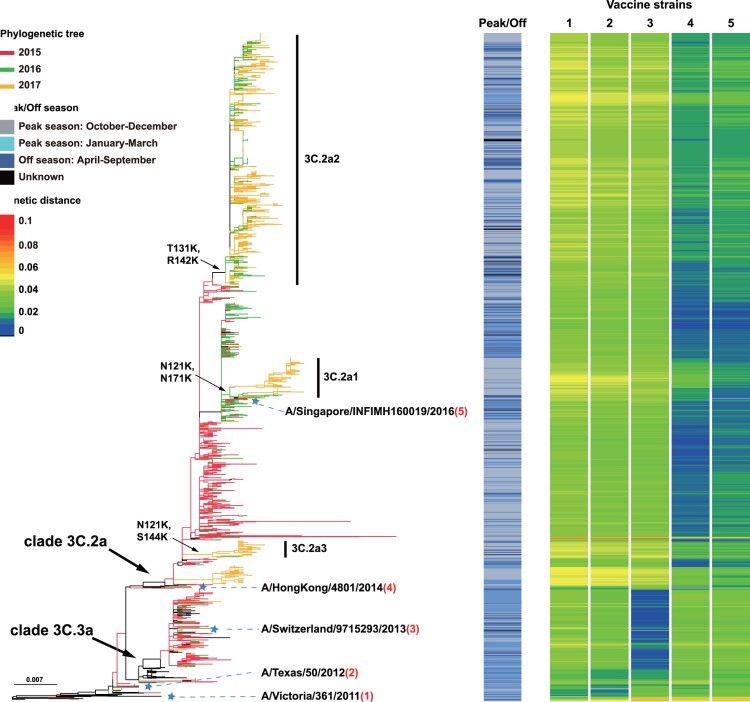
Genetic distances of the HA1 protein between egg-propagated vaccine strains and the circulating strains from China. The left part shows the phylogenetic tree of the HA gene sequences of the Chinese strains sampled since 2015.The five vaccine strains proposed by the WHO are highlighted in the tree and numbered with 1–5. The right panel shows the genetic distances of the HA1 protein between egg-propagated vaccine strains and the circulating strains from China, estimated using the full-length HA1 protein sequences using Phylip (Felsenstein 2004). The numbers 1–5 in the upper right corner correspond to the five vaccine strains shown in the tree in the left panel.

**Figure 4.**
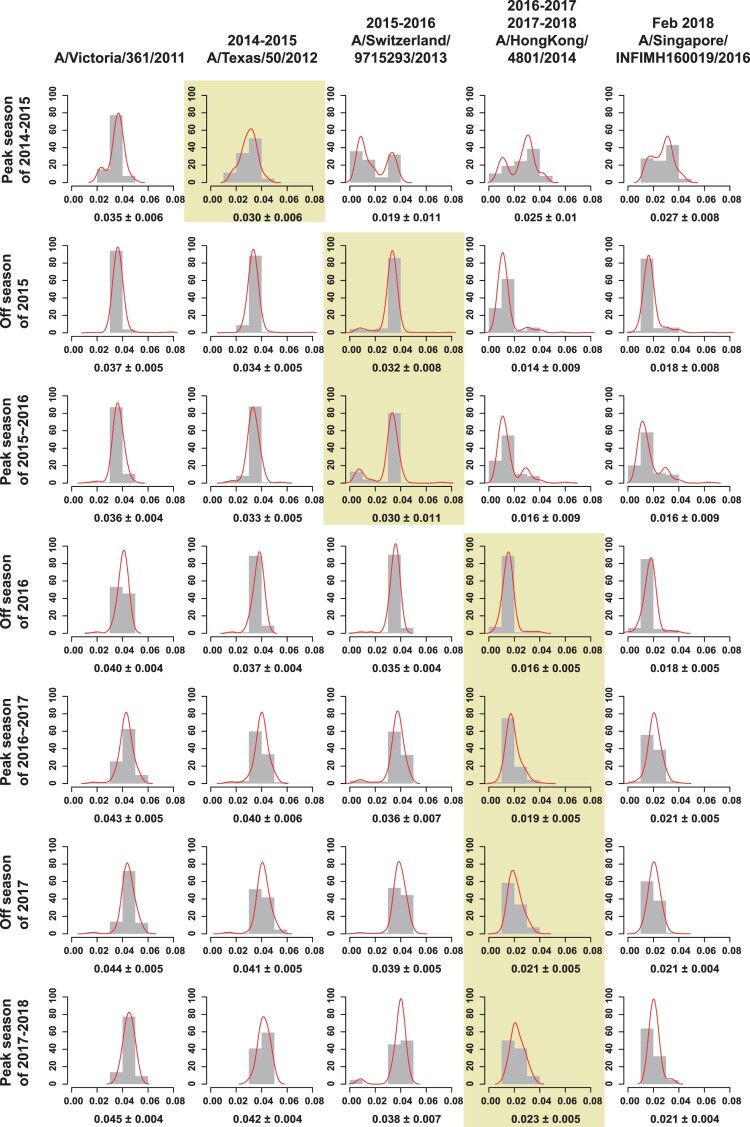
The average genetic distance between egg-propagated vaccine strains and the circulating strains from China across different influenza seasons. The grey bars show the proportion of genetic distances within each 0.01 interval with an increase by genetic distance of 0.01. The red lines are the Gaussian regression curve of the genetic distance. The shaded areas indicate the period of time when the vaccine was proposed to be used until the next vaccine strain is proposed.

The major epitopes of seasonal influenza viruses are located in the HA1 protein [23]. To help assess vaccine effectiveness and the optimal timing for vaccine strain updates, we estimated the distances between the vaccine strains and the Chinese strains since 2015 using the full-length HA1 protein sequences (Figures 3 and 4). A/Texas/50/2012 was recommended by World Health Organization (WHO) to be the vaccine candidate strains for the 2014–2015 influenza season in the northern hemisphere, while A/Switzerland/9715293/2013 was the vaccine strain for the 2015 off-season and the 2015–2016 northern hemisphere influenza season. From the 2016 off-season until February 2018, A/HongKong/4801/2014 was the vaccine strain, when it was replaced by A/Singapore/INFIMH160019/2016 (http://www.who.int/influenza/vaccines/virus/recommendations/2018_19_north/en/).

Genetic distances between strains were visualized by a colour gradient from blue to red, with blue representing distance of 0 and red representing distance of 0.1 (Figure 3). Egg-propagated vaccine strains A/Victoria/361/2011 and A/Texas/50/2012 had similar distances to the Chinese strains during 2015–2017, with most distances > 0.03, especially for subclades 3C.2a1-3C.2a3 (Figure 3). A/Switzerland/9715293/2013 was highly similar to viruses in the 2015–2016 influenza season, especially those of subclade 3C.3a and revealed by dark blue colours. Generally, lower genetic distances were found between the 2017 circulating strains and A/HongKong/4801/2014 than A/Singapore/INFIMH160019/2016 (Figure 3). However, A/Singapore/INFIMH160019/2016 was more similar to the 121 K variants than A/HongKong/4801/2014, especially for 3C.2a1 (Figure 3). Similar results were obtained when using cell culture-propagated vaccine strains, although genetic distance values were generally smaller (data not shown).

Unfortunately, a delay in vaccine strain update was observed in China, whatever egg-propagated (Figure 4) or cell culture-propagated (Figure S2) vaccine strains were used in the calculations. The mean genetic distances between the circulating strains and the egg-propagated vaccine strain used contemporarily were ≥0.03 from the 2014–2015 to the 2015–2016 influenza season (Figure 4). In contrast, vaccine strains employed later had smaller distances with previously circulating strains. For example, the vaccine strain A/Switzerland/9715293/2013 used in the 2015–2016 influenza season had lower genetic distances with the circulating strains in the 2014–2015 season than the previous vaccine strain A/Texas/50/2012 - 0.019 vs 0.030 (Figure 4). Similarly, compared with A/Switzerland/9715293/2013, A/HongKong/4801/2014(the vaccine strain recommended in the 2016 off-season) had a smaller genetic distance of < 0.016 to the majority of the viruses from the 2015 off-season and the 2015–2016 influenza season (Figure 4). A delay of vaccine strain update was also observed in other regions in the northern hemisphere, such as North America (Figure S3) and Europe (Figure S4), regions in the southern hemisphere, such as Australia (Figure S5), and even in the tropics, such as Southeast Asia (Figure S6).

### Persistence of A/H3N2 in southern China

Although a small number of viruses with the 121 K mutation have been previously isolated in China, they did not cluster with the lineage 3C.2a (data not shown). In our analysis, a total of 153 Chinese strains possessed the 121 K mutation: 6 in genotype 3C.2a, 102 in 3C.2a1, 45 in 3C.2a2 and one in 3C.2a3. The earliest strain was A/Guangdong-Zhanjiang/SY486/2015, which was isolated on June 1, 2015. Since then, only 15 121 K strains were identified in China before the 2017 off-season in our routine surveillance, whereas 124 121 K variants were reported in the 2017 off-season.

**Figure 5.**
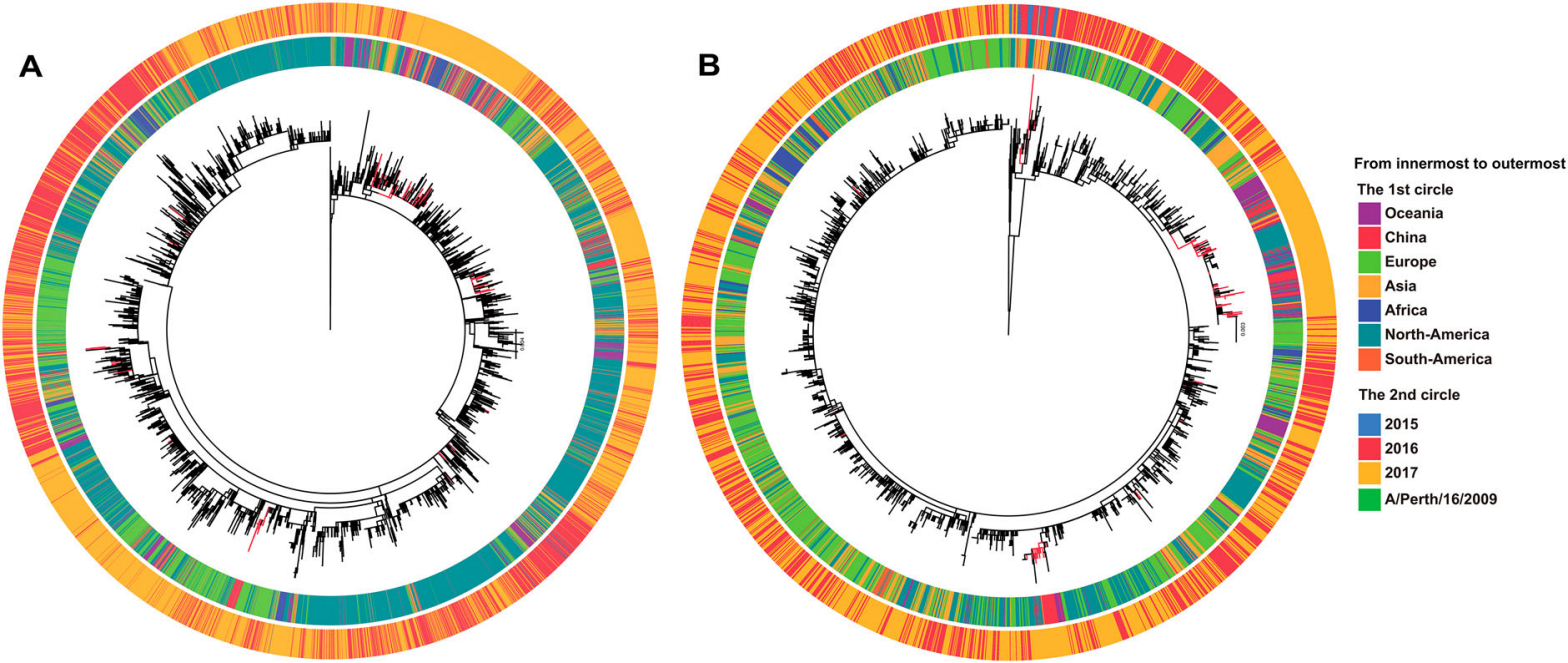
Phylogenetic analysis of the HA gene sequences of worldwide 121 K variants. Panel A: the 3C.2a1 subclade; panel B: the 3C.2a3subclade. The Chinese strains are highlighted in red in the two trees.

To investigate the origin of the Chinese 121 K variants, we performed an MDS analysis of the worldwide 121 K strains (*n* = 10,088). This indicated that they could be divided into two major groups (Figure S7) that generally corresponded to the two named 121 K subclades, 3C.2a1 and 3C.2a3, respectively. We then extracted the sequences of the two groups (3C.2a1, *n *= 7456; 3C.2a3, *n *= 2624) and performed separate phylogenetic analyses using RAxML (Figure 5 and supplementary files 3-4). This revealed that many Chinese 121 K variants were not located at the main trunk of the trees, but rather appeared as terminal branches (i.e. tips) (Figure 5). This suggests that the majority of the Chinese 121 K variants were imported from external sources multiple times, such as Europe, Australia and other Asian countries (Figure 5).

## Discussion

The influenza peak in the summer of 2017 (May-August) in southern China, especially in Hong Kong, received major attention due to the high hospitalization/fatality rate and the emergence of a novel A/H3N2 variant that possessed the 121 K mutation in the HA protein. Subsequently, China reported the second-highest recorded number of influenza cases in the 2017–2018 influenza season since the pandemic H1N1 influenza virus in 2009 [3]. To shed light on the circulation pattern of seasonal influenza in China, we analysed the surveillance data from the Chinese National Influenza Centre and 1417 full-length genome sequences of A/H3N2 influenza virus isolated from China since 2015.

Our surveillance data showed that since late 2014, A/H3N2 influenza peaks were observed in the 2014–2015 and 2016–2017 influenza seasons in northern China. In the 2015–2016 influenza season, A/H3N2 co-circulated with seasonal A/H1N1 and influenza B in northern China. In contrast, A/H3N2 predominated in the summer peaks of 2015 and 2017, as well as in the 2016–2017 influenza season in southern China. In addition, we further reported that even in winter months the dominant influenza subtype between southern and northern China was not always the same. In particular, a minor spring influenza peak caused by a virus different from the dominant subtype in winter was found. Together, these results suggest a heterogeneous and complex influenza circulation pattern in China which might be shaped by such factors as herd immunity and climatic variation.

Compared with influenza A/H1N1 and B, numerous studies have revealed that A/H3N2 experienced more rapid antigenic variation and genetic variants of A/H3N2 were reseeded from the East Asia and Southeast Asia (E-SE) regional circulation network [24]. In recent years, several novel A/H3N2 variants have emerged globally, such as 3C.2a and its decedents 3C.2a1 and 3C.2a3 [7]. In the 2014–2015 influenza season, 3C.3a was found to be dominant in China. However, 3C.2a became the dominant genotype in the subsequent off-season of 2015 and the 2015–2016 influenza season. Although 3C.2a1 and 3C.2a3 have been reported to have increasing incidence and have become the dominant subtypes in some regions in 2017, such as Hong Kong [10], 3C.2a2predominated in mainland China, suggesting a diversifying circulation pattern in China. Furthermore, viruses of 3C.2a2were more closely related to A/HongKong/4801/2014 than the proposed vaccine strain by WHO in February 2018, A/Singapore/INFIMH160019/2016, which mainly targeted clade 3C.2a1 viruses. Therefore, the efficacy of this vaccine strain update in China warrants further investigation because the prevalence of the 121 K variants in China was not high during our surveillance period.

The amino acid substitutions in the epitopes of the HA protein of human influenza viruses would likely result in changes in antigenicity, helping the viruses evade human immune responses. Our analysis revealed that the clades 3C.2a1 (N121 K and N171 K), 3C.2a3 (N121 K and T135 K), and 3C.2a2 (T131 K and R142 K) possessed amino acid substitutions in the known epitopes A or D of A/H3N2, and experimental data from recent reports have revealed antigen variation among these clades [25]. In particular, the two clades possessing the 121 K substitution - 3C.2a1 and 3C.2a3 - had reduced vaccine effectiveness [9]. Therefore, the proposed vaccine strain A/Singapore/INFIMH160019/2016 from clade 3C.2a1 might have higher vaccine efficacy to the 121 K variants than A/HongKong/4801/2014.

Attempting to predict the genetic and antigenic variation of human influenza has been one of the most important and controversial topics in influenza research [26] and several sequence-based machine learning methods have been proposed [6,27,28]. Such efforts could be potentially helpful for selection of appropriate candidate vaccine strain from circulating strains, which plays a pivotal role in the vaccine effectiveness. Unfortunately, our sequence-based analysis revealed a distinct delay (approximately one year later) of the A/H3N2 vaccine strain update in China, which has also been noted previously [6]. Similar delays have been observed in other regions, such as North America, Europe and Australia. Although our analysis was sequence-based and this might not reflect the real antigenic relationships between vaccine and circulating strains, considering the fact that the influenza vaccine effectiveness was not high in both China [3] and the USA [29], we believe that a novel and more efficient vaccine selection strategy is urgently required to account for the different influenza circulation patterns across continents and even major populous countries.

Apart from the delayed vaccine strain update, the egg-adaptive mutations occurring during the egg passage of the A/H3N2 vaccine seed strains would alter glycosylation and impair the neutralizing antibody response, resulting in reduced vaccine effectiveness [30]. For example, the L194P mutation on influenza HA was one of the most commonly observed egg-adaptive mutations and would significantly alter HA antigenicity [31]. A recent study reported that introduction of theG186 V mutation would only minimally alter HA antigenicity, but prevent the occurrence of L194P by means of mutationalincompatibility [31].

Influenza infection rates show clear seasonal variation and the peak of influenza season is concentrated in winter in temperate regions, usually between October and March in the northern hemisphere, the so-called peak season, whereas the remaining months are called often termed the off-season [32]. However, in the tropics, influenza infection remains at low levels throughout the year, with a peak often during the rainy season [33,34]. Despite the climatic variation, a recent analysis of the influenza surveillance data of Australia during 2007–2016 revealed distinct continental synchronicity of human influenza virus epidemics [35]. To date, a number of models have been proposed to account for the seasonality of seasonal influenza, such as global migration [5,36,37] and both global [38] and local persistence [39]. We found that the 121 K variant was seldom isolated in 2016, while most of the Chinese 121 K variants were observed in the summer of 2017 in southern China. Combined with the phylogenetic analysis, we suggest that these variants were imported from external sources to China multiple times. A previous study based on the ILI surveillance data during 15 years in Shenzhen, southern China also revealed multiple viral introductions [40]. Both studies suggest that China, even southern China, could also be a sink region in the global persistence model of human A/H3N2 influenza virus.

In sum, our national surveillance of ILI reveals the complex patters of circulation of different influenza subtypes and the co-circulation of multiple A/H3N2 3C.2a derived variants with minor antigenic variations in China. Not only do these data reveal the difficulties in vaccine strain selection, which clearly merit further investigation, but our analyses highlight the multiple introductions and circulation of the 121 K variants in this country as well as the worrying delay in vaccine strain updates both in China and globally.

## Supplementary Material

Supplemental Material
