# Supplementary material for: Co-circulation and persistence of multiple A/H3N2 influenza variants in China
Source: Emerg Microbes Infect. 2019 Aug 2;8(1):1157–67. doi: 10.1080/22221751.2019.1648183 (PMC6713139; doi:10.1080/22221751.2019.1648183)
Supplement: Supplemental Material [file TEMI_A_1648183_SM8628.zip › Table S1. 20190618_final.doc]

| Collection date | | Clade 3C | Clade 3C.1 | Clade 3C.2 | Clade 3C.2a | Clade 3C.2b | Clade 3C.3 | Total |
| --- | --- | --- | --- | --- | --- | --- | --- | --- |
| 2014-2015Peak season | Southern China | 19 |  | 23 | 30 |  | 69 | 141 |
|  | Northern China | 4 |  |  | 2 |  | 18 | 24 |
| 2015Off-season | Southern China | 2 |  | 5 | 182 | 21 | 17 | 227 |
|  | Northern China |  |  |  | 9 | 2 | 1 | 12 |
| 2015-2016Peak season | Southern China | 3 | 2 | 3 | 23 | 21 | 15 | 67 |
|  | Northern China |  |  |  | 43 | 102 | 26 | 171 |
| 2016Off-season | Southern China | 1 |  | 1 | 8 | 58 | 1 | 69 |
|  | Northern China |  |  |  |  | 8 |  | 8 |
| 2016-2017Peak season | Southern China | 4 | 4 | 2 | 4 | 273 | 14 | 301 |
|  | Northern China |  |  |  | 1 | 55 | 4 | 60 |
| 2017Off-season | Southern China | 2 | 5 |  | 44 | 393 | 6 | 450 |
|  | Northern China |  |  |  | 1 | 9 |  | 10 |
| 2017-2018Peak season | Southern China |  |  |  | 3 | 6 |  | 9 |
|  | Northern China |  |  |  | 7 | 5 | 1 | 13 |
| Total |  | 35 | 11 | 34 | 357 | 953 | 172 | 1562 |

Table S1. Temporal distribution of the NA genotypes of influenza A/H3N2 virus in China
